# Arabidopsis MED18 Interaction With RNA Pol IV and V Subunit NRPD2a in Transcriptional Regulation of Plant Immune Responses

**DOI:** 10.3389/fpls.2021.692036

**Published:** 2021-10-06

**Authors:** Yan Zhang, Chengchen Shi, Weihong Fu, Xiaojing Gu, Ziyang Qi, Weizhong Xu, Gengshou Xia

**Affiliations:** ^1^Department of Landscape and Horticulture, Ecology College, Lishui University, Lishui, China; ^2^Lishui Academy of Agricultural and Forestry Sciences, Lishui, China

**Keywords:** Mediator, MED18, NRPD2a, RNA polymerase IV and V, plant immunity, epigenetic regulation, necrotrophic pathogens, *Botrytis*

## Abstract

Mediator is a conserved multiprotein complex important for transcription by RNA polymerase II (Pol II). Arabidopsis Mediator subunit MED18 regulates flowering, hormone signaling and plant immunity. Here we report that Arabidopsis MED18 interacted with NUCLEAR RNA POLYMERASE D2a (NRPD2a), the second largest subunit of the nuclear Pol IV and V, which function in RNA-directed DNA methylation and epigenetic regulation of gene expression. Mutants for both *MED18* and *NRPD2a* were compromised in resistance to necrotrophic fungal pathogen *Botrytis cinerea*. Mutants for *NRPD1a*, the largest subunit of Pol IV, were also compromised in resistance to *Botrytis*, supporting a critical role of Pol IV and V in plant defense against *Botrytis*. Increased *Botrytis* susceptibility of both the *med18* and *nrpd2a* mutants were associated with reduced accumulation of reactive oxygen species, which are known to promote resistance to *Botrytis*. Both the basal and pathogen-induced levels of salicylic acid and jasmonic acid were also significantly altered in the *med18* and *nrpd2a* mutants. Transcriptome profiling found that MED18 and NRPD2a affected both unique and overlapping sets of genes in a broad spectrum of biological processes and pathways that influence plant–pathogen interaction. The genes altered in expression in the *med18* and *nrpd2a* mutants include disease resistance proteins, salicylic acid and jasmonic acid signaling and responses, which are known to affect resistance to necrotrophic pathogens. The novel interaction between subunits of Mediator and plant-specific RNA polymerases provides a new mechanism for epigenetic regulation of resistance and expression of defense-related genes in plant immunity.

## Introduction

Plants are constantly exposed to a variety of microbial pathogens with different virulence mechanisms and have evolved a multi-layered immunity system with complex sets of infection-induced defense mechanisms (Jones and Dangl, 2006). The first layer of infection-induced defense mechanisms is triggered upon recognition of pathogen-associated molecular patterns (PAMPs) by plant plasma membrane-localized pattern recognition receptors (PRRs). Adapted biotrophic and hemibiotrophic pathogens deliver effector proteins to plant cells to suppress PAMP-triggered immunity (PTI) (Dou and Zhou, 2012). To counter this, plants have evolved the second layer of the immunity system through recognition of pathogen effectors to activate effector-triggered immunity (ETI), which is often associated with the rapid activation of hypersensitive cell death and increased biosynthesis of salicylic acid (SA). Necrotrophic pathogens, on the other hand, kill host cells before colonizing them (Mengiste, 2012). Hypersensitive cell death is effective against biotrophic pathogens but facilitate the infection and, therefore, is promoted by necrotrophic pathogens through toxic secondary metabolites, small secreted proteins, and small RNAs (Mengiste, 2012). In Arabidopsis, resistance to necrotrophic pathogens depends on jasmonate (JA) and ethylene (ET) signaling and synthesis of the phytoalexin camalexin (Mengiste, 2012). Activation of complex plant defense mechanisms is associated with diverse signaling processes including dynamic protein interactions and phosphorylation, generation of reactive oxygen species, Ca^2+^ signal spike and mitogen-activated protein kinase (MAPK) activation (Tsuda and Katagiri, 2010). These signaling processes then often converge in the nucleus to elicit host transcriptional reprogramming not only for defense but also for balancing plant defense and fitness.

Mediator is a conserved multi-protein complex that plays an important role in regulating transcription by mediating interactions between transcriptional activator proteins and RNA polymerase II (Pol II) (Conaway and Conaway, 2011). The Mediator complex contains more than 30 subunits and comprises four distinct modules termed the head, middle, tail, and cyclin-dependent kinase 8 (CDK8) module that is reversibly linked with Mediator (Conaway and Conaway, 2011). Even though Mediator plays a constitutive role in the transcription of all genes transcribed by Pol II, specific Mediator subunits may control the developmental and environmental regulation of specific subsets of Pol II-dependent genes (Poss et al., 2013). In Arabidopsis, Mediator subunits play an important role in the regulation of development, flowering (Inigo et al., 2012; Jaskolowski et al., 2019), non-coding RNA processing (Kim et al., 2011), secondary metabolism, and tolerance to abiotic stresses and phytohormone signaling (Elfving et al., 2011; Bonawitz et al., 2012). Plant Mediator subunits are also involved in plant immunity, which relies on the crosstalk and balance in signaling of important defense-related phytohormones such as SA, JA, and ET against pathogens with distinct virulence mechanisms (An and Mou, 2013). SA signaling generally triggers resistance against biotrophic and hemibiotropic pathogens, while JA and ET signaling usually activates resistance against necrotrophic pathogens. SA- and JA/ET-dependent signaling pathways often antagonize each other (Mengiste, 2012). A number of plant Mediator subunits regulate SA- or JA-dependent expression of defense-related genes and resistance to biotrophic or necrotrophic pathogens. Arabidopsis MED25 is an important component in JA-dependent defense gene expression and resistance to necrotrophic fungal pathogens *Alternaria brassicicola* and *Botrytis* (Kidd et al., 2009). Conversely, MED25 negatively regulates resistance to *Fusarium oxysporum*, a root-infecting hemibiotrophic fungal pathogen, which apparently uses the host JA pathway to promote host senescence and necrosis (Thatcher et al., 2009). Similar to MED25, MED8 plays a positive role in resistance to *A. brassicicola* but a negative role in resistance to *F. oxysporum* in Arabidopsis, suggesting that MED8 also mediates JA-dependent defense (Kidd et al., 2009). Consistent with their similar roles, the *med8* mutation has an additive effect with the *med25* mutation on *F. oxysporum* resistance (Kidd et al., 2009). On the other hand, Arabidopsis MED15 plays a role in the regulation of SA response. Arabidopsis mutants for MED15 are insensitive to benzol (1,2,3) thiadiazole- 7-carbothioic acid S-methyl ester (BTH), an analog of SA and are also compromised in biological induction of SA-mediated systemic acquired resistance (An and Mou, 2013). Likewise, MED16 is a key positive regulator of SAR and is required for SAR-associated defense gene expression (Zhang et al., 2012).

Arabidopsis MED18 is a multifunctional Mediator subunit that affects flowering, hormone signaling and plant immunity through interactions with multiple transcription factors (Lai et al., 2014; Liao et al., 2016). In the present study, we discovered that Arabidopsis MED18 interacted with NRPD2a, the second largest subunit of the nuclear RNA polymerase IV and V (Pol IV and V) involved in epigenetic regulation of gene expression (Ream et al., 2014). Mutants for MED18, NRPD2a and NRPD1a, the largest subunit of Pol IV were all compromised in resistance to necrotrophic fungal pathogen *Botrytis*. We also performed transcriptome profiling of Arabidopsis wild-type (WT), *med18* and *nrpd2a* mutants and found that MED18 and NRPD2a affected both unique and overlapping sets of genes involved in defense responses, plant-pathogen interactions, secondary metabolism, hormone signaling and stress responses. Defense-related genes coregulated by MED18 and NRPD2a also included those encoding pathogenesis-related proteins, disease resistance proteins and SA biosynthetic enzymes, which are known to promote susceptibility to necrotrophic pathogens such as *Botrytis*. These results further support a critical role of epigenetic regulation of defense-related genes in plant immunity.

## Materials and Methods

### Plant Materials and Growth Condition

The Arabidopsis plants used in this study are in the Col-0 background. The *med18-1* (Sail-889-C08), *nrpd1a-1* (SALK_143437C), *nrpd1a-3* (SALK_128428), and *nrpd2a-2* (SALK_046208) mutants have been previously described (Li et al., 2006; Lopez et al., 2011; Lai et al., 2014). The *nrpd2a-3* (WiscDsLoxHs048_04C) mutant was obtained from Arabidopsis Resource Center at the Ohio State University and homozygous lines were identified by PCR using gene-specific primers flanking the insertion sites (Supplementary Table 1 and Supplementary Figure 1A). RT- qPCR analysis indicated that the *nrpd2a-3* mutant is a knockout mutant with little *NRPD2a* transcript detected (Supplementary Figure 1B). Arabidopsis plants were grown in a growth chamber at 22 ± 2°C under 200 μmol m^–2^ s^–1^ light with a 12 h light/12 h dark cycle.

### Yeast Two-Hybrid Screens

In order to find MED18-interacting proteins, Gal4 based yeast-two-hybrid system was utilized as previously described (Lai et al., 2011). Briefly, *MED18* coding sequence was PCR-amplified using gene specific primers (Supplementary Table 1) and cloned into pBD-GAL4 vector to generate the bait vector. The Arabidopsis HybridZAP-2.1 two-hybrid cDNA library was prepared from Arabidopsis plants. The bait plasmid and the cDNA library were used to transform yeast strain YRG-2. Yeast transformants were plated onto selection medium lacking Trp, Leu, His, and confirmed by β-galactosidase activity assays using X-gal as substrate.

### Bimolecular Fluorescence Complementation

Vectors for Bimolecular Fluorescence Complementation (BiFC) (pFGC-N-YFP and pFGC-C-YFP) were previously described (Kim et al., 2008). Full-length coding sequence of *MED18* was inserted into pFGC-C-YFP and NRPD2a, NRPD2aCTD, and NRPD2aNTD coding sequences were inserted into pFGC-N-YFP (Supplementary Table 1). The fusion constructs were introduced into *Agrobacterium tumefaciens* (strain GV3101) and infiltrated into *N. benthamiana* leaves as described previously (Lai et al., 2014). BiFC signals in the infiltrated leaf tissues were examined 48 h after infiltration with a Zeiss LSM710 confocal microscope and images were superimposed with Zeiss LSM710 software.

### Coimmunoprecipitation Assays

The HA-tagged MED18 fusion construct (MED18-3xHA) under control of CaMV 35S promoter in a modified version of binary vector pCAMBIA99-1 has been previously described (Lai et al., 2014). The *NRPD2a* coding sequence was fused with the MYC tag (NRPD2a-6xMYC) and cloned into binary vector pBA-Myc under 35S promoter (Supplementary Table 1). These constructs were introduced into *A. tumefaciens* strain GV3101 and transformed into Arabidopsis using the floral dip method (Clough and Bent, 1998). Arabidopsis plants expressing both the MED18 and NRPD2a constructs were generated through genetic crossing. Proteins were extracted in an extraction buffer [50 mM Tris-HCl, pH7.5, 100 mM NaCl; 2 mM EDTA; 1 mM NaF; 1 mM NaVO3; 1 mM PMSF; 10 mM *b*-glycero phosphate; 0.1% (v/v) Triton X-100; 0.5% (v/v) Nonidet P-40; and 1x protease inhibitor cocktail]. After centrifugation, the supernatant was incubated with anti-HA-conjugated agarose beads (Sigma-Aldrich) for 12 h at 4°C. The Coimmunoprecipitation (Co-IP) products were washed with the extraction buffer four times and then detected by protein blotting.

### Generation of Transgenic NRPD2a-Overexpression Lines

The full-length coding sequence of *NRPD2a* was PCR-amplified using gene-specific primers (Supplementary Table 1) and cloned behind the *CaMV 35S* promoter in the binary vector pCAMBIA120. The construct was introduced into *A. tumefaciens* strain GV3101 and transformed into Arabidopsis using the floral dip method (Clough and Bent, 1998). Total RNA and cDNA were prepared as previously described (Zhang et al., 2021). The transgenic lines overexpressing NRPD2a were identified by reverse transcription - PCR (RT-PCR) using gene -specific primers (Supplementary Table 1 and Supplementary Figure 2). T2 homologous lines were used in the assays of resistance to *Botrytis*.

### Pathogen Inoculation and Disease Resistance Assays

*Botrytis* inoculation was performed by spraying on whole plants with a spore suspension (2 × 10^5^ spores per ml) in Sabouraud maltose broth buffer. In both cases, inoculated plants were kept under a transparent cover to maintain high humidity. Fungal biomass in inoculated plants was quantified by RT-PCR using primers specific to *Botrytis* Actin gene as previously described (Lai et al., 2014).

Inoculation of the bacterial pathogen *Pseudomonas syringae* pv *tomato* DC3000 by leaf infiltration was performed as previously described (Wang et al., 2014). Inoculated leaves were homogenized in 10 mM MgCl_2_ and diluted before plating on King’s B Agar with 25 μg/ml rifampicin. Colony forming units were determined 2 days after bacteria growth at 28°C.

### RT-qPCR Analysis of Gene Expression

Total RNA and cDNA were prepared as described above. Real time-quantitative - PCR (RT-qPCR) was performed using the CFX96 Touch^TM^ real-time PCR detection system (Bio-Rad, CA, United States) and SYBR Premix Ex TaqTM kits (TaKaRa, Dalian, China) with gene-specific primers (Supplementary Table 1).

### Histochemical Staining of Cell Death and Reactive Oxygen Species

The histochemical staining of *Botrytis*-induced cell death, superoxide and hydrogen peroxide using trphan blue, nitroble tetrazolium (NTB) and 3,3′-diaminobenzidine (DAB), respectively, were performed as previously described (Jabs et al., 1996; Thordal-Christensen et al., 1997; van Wees, 2008).

### Salicylic Acid and Jasmonate Quantification

The endogenous SA and JA were extracted following the method (Zhang et al., 2018) from Arabidopsis leaves with D4-SA and D5-JA as internal standards. And the contents were identified by LC-MS/MS (AD30-Qtrao6500, Sciex) according to the method (Wang et al., 2016).

### Total RNA Isolation, Library Construction, and RNA-seq

Total RNA was isolated from Arabidopsis plants using Trizol reagent (Sigma, United States), according to the manufacturer’s instructions. Genomic DNA was removed with DNaseI (RNase-free) (NEB, United States). RNA purity, concentration and integrity were confirmed using Nanodrop (Thermo Scientific, United States), Qubit 2.0 fluorometer (Thermo Scientific, United States) and Agilent 2100 Bioanalyzer (Agilent, United States), respectively.

For construction of cDNA library, mRNA was isolated from total RNA using poly(dT) oligo-attached magnetic beads. First-strand cDNA was synthesized from fragmented mRNA using random primers. Second strand cDNA was synthesized using RNase H and DNA polymerase I. Double-strand cDNA were purified by AMPure XP beads and the cDNA library was constructed after PCR enrichment. Three biological replicates were used per condition for each genotype. Samples were subjected to high throughput sequencing using Illumina HiSeq2500.

### Quantification of Transcripts, Identification of Differentially Expressed Genes, and Other Bioinformatic Analyses

The adapter sequences from the RNA-seq data were trimmed and low-quality reads were removed from raw data to gain clean reads. Clean reads were aligned to Arabidopsis genome TAIR10 (Lamesch et al., 2012) to obtain and improve mapped reads using TopHat2 (Kim et al., 2013) and Bowtie (Langmead et al., 2009) software, respectively. BLAST (Altschul et al., 1997) was used to obtain annotations for new genes using data bank NR (Apweiler et al., 2004), Swiss-Prot (Ashburner et al., 2000), GO (Tatusov et al., 2000), COG (Koonin et al., 2004), KOG (Finn et al., 2014), Pfam (Kanehisa et al., 2004), and kyoto encyclopediaof genes and genomes (KEGG) (Kanehisa et al., 2004).

Read Per Kilobase of transcript per Million fragments mapped (RPKM) (Anders and Huber, 2010) was calculated by Cuffdiff to measure gene expression quantity. Fold-Change ≥ 2, Fold-Change ≤ 0.5 and False Discovery Rate (FDR) < 0.01 were used as standard to identify Differentially Expressed Gene (DEG) by DESeq (Kanehisa et al., 2004). Pearson’s Correlation Coefficient (*r*^2^) was used as evaluation index for biological replicates.

Principal component analysis (PCA)was performed using R studio with ggplot2 package. AgriGO was used to determine gene ontology (GO) enrichment (Du et al., 2010). The ShinoGO v0.61 web tool^1^ was used to identify KEGG pathways (Ge et al., 2020). Venn diagrams were generated displaying the DEGs with FDR < 0.05 for comparisons between groups of samples using Venny^2^.

## Results

### Identification of NRPD2a as an Interacting Protein of MED18 From Y2H

**FIGURE 1:**
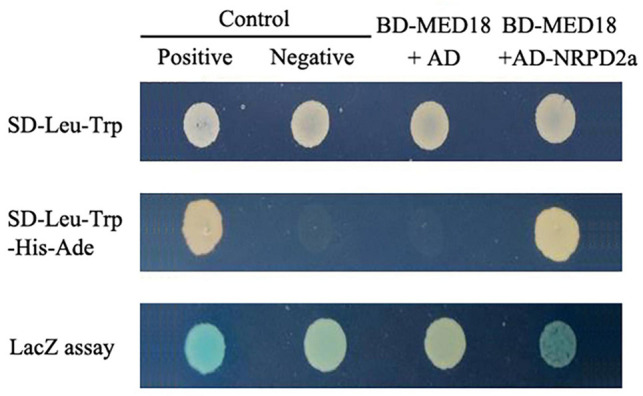
Yeast two-hybrid assays of MED18-NRPD2a interaction. A vector containing Gal4 DNA-binding domain (BD) fused with MED18 (BD-MED18) was cotransformed with a Gail4 activation domain (AD) empty vector (AD) or AD-NRPD2a fusion vector into yeast cells. For comparison, control vectors containing BD and AD with (Positive) or without (Negative) fusion with self-interacting cI fragment from lambda phage were also cotransformed into yeast cells. The transformed yeast cells were grown on the selection medium with (SD-Leu-Trp) or without His and Ade (SD-Leu-Trp-His-Ade). Transformed yeast cells were also assayed for β-galactosidase (LacZ) activity using X-gal as substrate.

Arabidopsis MED18 is a subunit of the mediator complex that affects plant immunity, flowering time, floral organ formation and responses to hormones (Lai et al., 2014). The multifunctional roles of MED18 are mediated through its interaction with distinct transcriptional regulators including YIN YANG1, ABA INSENSITIVE 4, SUPPRESSOR OF FRIGIDA4, and HOOKLESS1 (Lai et al., 2014; Liao et al., 2016). To gain further insights into the action of MED18, we tried to identify additional MED18-interacting proteins using Gal4-based yeast two-hybrid screens with MED18 as a bait. After screening 2 × 10^6^ independent transformants of an Arabidopsis cDNA prey library, we isolated only one positive clone based on both prototrophy for His and LacZ reporter gene expression through assays of β-galactosidase activity. The clone identified from the screens encode NUCLEAR RNA POLYMERASE D2 (NRPD2a, AT3G23780), the second largest, catalytic subunit of the nuclear DNA-dependent RNA polymerase IV and V (RNA Pol IV and V). As shown in Figure 1, yeast cells cotransformed with both the pBD-MED18 bait and pAD-NRPD2a prey vector had a high level of β-galactosidase activity similar to that of the positive control. By contrast, little β-galactosidase activity was detected in the yeast cells transformed with the pBD-MED18 bait vector and the pAD empty vector (Figure 1).

### Subcellular Localization of MED18-NRPD2a Interaction by Bimolecular Fluorescence Complementation

**FIGURE 2:**
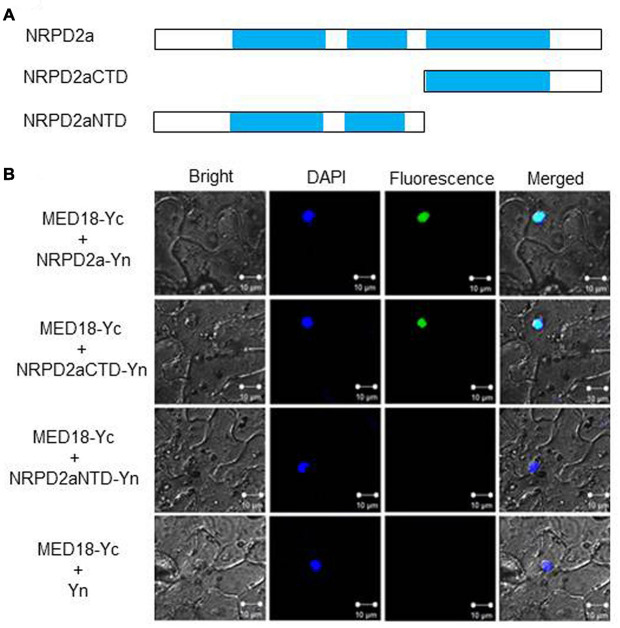
BiFC assays of MED18-NRPD2a interaction. **(A)** Diagrams of NRPD2a full-length protein (NARPD2a), C-terminal (NARPD2aCTD)and N-terminal domain (NARPD2aNTD). **(B)** BiFC signals from interaction of MED18-NRPD2a interaction. Bright-field, DAPI staining, YFP epifluorescence and emerged images of the same cells are shown. Bar = 10 μm.

To determine whether MED18 and NRPD2a interact in plant cells, we performed BiFC in *Agrobacterium*-infiltrated *Nicotiana benthamiana*. We fused Arabidopsis MED18 to the C-terminal yellow fluorescent protein (YFP) fragment (MED18-Yc) and fused the full-length NRPD2a to the N-terminal YFP fragment (NRPD2a-Yn). NRPD2a is a protein of 1172 amino acid residues. The N-terminal half of NRPD2a contains RNA polymerase Rpb2 domains 2 and 3, whereas the C-terminal half contains the catalytic center including the binding site for magnesium ions that guide free nucleoside triphosphates into the active site for RNA synthesis, stabilize the transition state of the growing RNA chain and participate in transcript cleavage events during polymerase backtracking, a process which helps prevent polymerase arrest at pause sites (Ream et al., 2014). In order to identify the region of NRPD2a responsible for interaction with MED18, we also fused both the N- and C-terminal halves to the N-terminal YFP fragment to generate NRPD2aNTD-Yn and NRPD2aCTD-Yn, respectively (Figure 2A). MED18-Yc was co-expressed with the full-length NRPD2a-Yn, truncated NRPD2aNTD-Yn or NRPD2aCTD-Yn in the leaves of *N. benthamiana*. As control, we also included in the assay the MED18-Yc construct coexpressed with the N-terminal YFP fragment (Yn). As shown in Figure 2B, complementation of MED18-Yc with the full-length NRPD2a-Yn generated BiFC signals in tobacco cells that overlapped with the 4′,6′-diamidino-2-phenylindole (DAPI) staining signals. This result indicated that interaction between MED18 and NRPD2a occurs in the nucleus. Reconstructed BiFC signals in the nucleus were also observed when MED18-Yc was coexpressed with NRPD2aCTD-Yn (Figure 2B). By contrast, no BiFC signal was observed when MED18-Yc was coexpressed with NRPD2aNTD-Yn or with unfused Yn empty construct (Figure 2B). Therefore, the C-terminal domain of NRPD2a is responsible for interaction with MED18.

### Confirmation of MED18-NRPD2a Interaction by Coimmunoprecipitation

**FIGURE 3:**
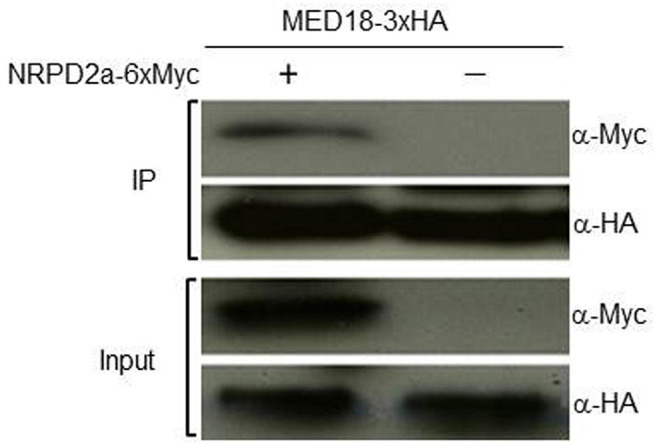
Co-IP assays of MED180NRPD2a interaction. Proteins were prepared from Arabidopsis plants expressing MED18-3xHA and NRPD2a-6xMYC constructs and subjected to co-IP using a-HA-tagged beads. Both input and IP proteins were analyzed with protein blotting using indicated antibodies.

To further confirm the MED18-NRPD2a interaction in the nucleus, we performed Co-IP to determine whether tagged MED18 complexes contained NRPD2a. First, we generated the epitope HA-tagged MED18 (MED18-3xHA) and MYC-tagged NRPD2a (NRPD2a-6xMYC) expression constructs and transformed them into Arabidopsis plants. An empty MYC tag vector was also transformed into Arabidopsis as control. Transgenic Arabidopsis co-expressing MED18-3xHA with NRPD2a-6xMYC or the empty 6xMYC construct were generated through genetic crosses. The nuclei were isolated from the seedlings of the transgenic plants and the nuclear protein extracts were subjected to immunoprecipitation using anti-HA antibody. Both inputs and immunoprecipitation fractions (IP a-HA) were subjected to immunoblotting with anti-HA (a-HA) or anti-MYC (a-MYC) antibodies. As shown in Figure 3, the NRPD2a-6xMYC could be co-immunoprecipitated with anti-HA antibodies when co-expressed with MED18-3xHA. No Co-IP signals were detected when MED18-3xHA was co-expressed with the empty 6xMYC construct (Figure 3). These results further confirmed that MED18 and NRPD2a interact in the nucleus of plant cells.

### Expression of *MED18* and *NRPD2a* in Response to *Botrytis* Infection

**FIGURE 4:**
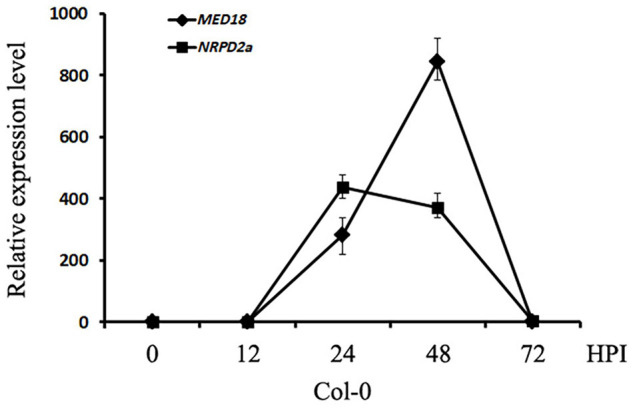
Expression of *MED18* and *NRPD2a* in response to *Botrytis* infection. Col-0 plants were inoculated with *Botrytis* and inoculated leaves were collected at indicated hours post inoculation for total RNA isolation. The relative transcript levels of *MED18* and *NRPD2a* were determined using RT-qPCR using Arabidopsis *ACTIN2* as internal control with gene-specific primers. The data represent mean ± SD from three biological replicates. The experiment has been repeated twice with similar results.

To determine whether MED18-interacting NRPD2a is involved in plant resistance to the necrotrophic fungal pathogen *Botrytis*, we compared the expression of both *MED18* and *NRPD2a* in response to infection by the fungal pathogen. As shown in Figure 4, both *MED18* and *NRPD2a* were rapid and strongly induced by Botrytis infection. At 24 h post inoculation (HPI), the transcript levels for *MED18* and *NRPD2a* were elevated by more than 200- and 400-fold, respectively. The transcript levels for *MED18* continued to increase during the next 24 h but then rapidly decreased to the basal levels by 72 HPI (Figure 4). The transcript levels for *NRPD2a* reduced slightly during the second day post inoculation before rapid decline to the basal levels by 72 HPI (Figure 4). The rapid and strong induction of *MED18* and *NRPD2a* by *Botrytis* infection strongly suggest their involvement in plant disease resistance.

### Functional Analysis of MED18 and Pol IV Subunits in Plant Disease Resistance

**FIGURE 5:**
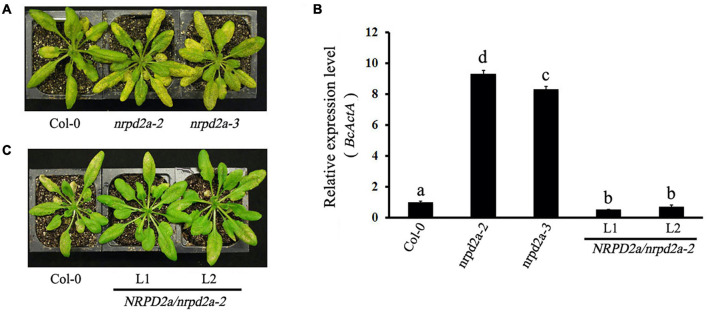
Altered responses of *nrpd2a* mutants and *NRPD2a* overexpression lines to *Botrytis*. **(A)** Disease symptoms of sprayed-inoculated Col-0 and *nrpd2a* mutants at 4 DPI. **(B)** Disease symptoms of sprayed-inoculated Col-0 and *NRPD2a* overexpression lines 1 (L1) and 2 (L2) at 4 DPI. **(C)** Accumulation of the *Botrytis BcActA* transcripts in sprayed-inoculated plants at 4 DPI. The data represent mean ± SD from three biological replicates. The experiments were repeated twice with similar results. Different letters are indicate significance of difference.

To determine directly the role of MED18-interacting NRPD2a in plant resistance to the necrotrophic fungal pathogen *Botrytis*, two T-DNA insertion mutants for NRPD2a (*nrpd2a-2* and *nrpd2a-3*) were compared with both Col-0 WT for responses to *Botrytis* infection. We also generated stable transgenic Arabidopsis plants that overexpressed NRPD2a under the control of the strong *CaMV 35S* promoter. Two independent transgenic overexpression lines with elevated levels of *NRPD2a* transcripts were identified by RT-qPCR (Supplementary Figure 2) and also compared with WT plants for responses to *Botrytis*. WT, mutant and overexpression plants were spray-inoculated with the fungal pathogen and observed for both symptom development and fungal growth. As shown in Figure 5A, at 4 days post inoculation (DPI), WT plants developed only small necrotic spots and chlorosis that did not spread significantly to cause extensive tissue damage. As a result, the majority of leaves from WT plants remained green at 4 DPI. In the *nrpd2a* mutants, the necrotic spots and chlorosis were very extensive, particularly in fully expanded leaves (Figure 5A). The transgenic *NRPD2a*-overexpressing plants, on the other hand, developed less chlorotic and necrotic symptoms than WT plants (Figure 5B).

To determine whether altered disease symptoms were correlated with pathogen growth in the mutants and overexpression lines for *NRPD2a*, we analyzed accumulation of the *Botrytis ActinA* (*BcActA*) gene transcript as a measure of fungal growth in inoculated plants. The transcript levels of the constitutively expressed *BcActA* gene correlated with fungal biomass. Total RNA was isolated from infected plants at 4 DPI, and quantified using RT-qPCR. As shown in Figure 4, substantially higher levels of the fungal *BcActA* gene transcript were detected in *nrpd2a* mutant plants than in WT plants after *Botrytis* infection. RT-qPCR analysis also showed reduced transcript levels of the constitutively expressed *BcActA* gene in the transgenic NRPD2a-overexpressing lines than in WT plants after *Botrytis* infection (Figure 5C). Thus, mutations of NRPD2a increased whereas its overexpression reduced both symptom development and fungal growth in the transgenic lines.

**FIGURE 6:**
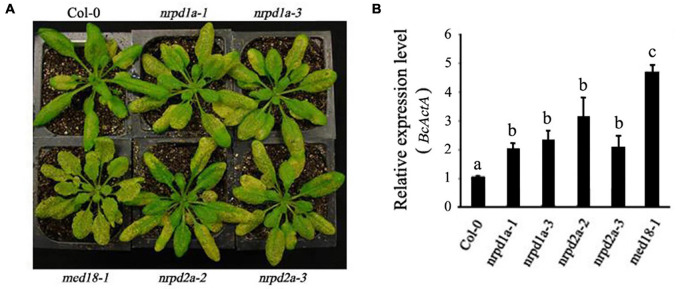
Altered responses of *med18*, *nrpd1a*, and *nrpd2a* mutants to *Botrytis*. **(A)** Disease symptoms of sprayed-inoculated plants at 4 DPI. **(B)** Accumulation of the *Botrytis BcActA* transcripts in sprayed-inoculated plants at 4 DPI. The data represent mean ± SD from three biological replicates. The experiments were repeated twice with similar results. Different letters are indicate significance of difference.

To determine shared biological functions of the MED18-NRPD2a interacting partners, we also compared the mutants for both transcription factors for resistance to the necrotrophic fungal pathogen. Unlike WT plants, the majority of leaves from the *med18* mutant were significantly macerated at 4 DPI (Figure 6A). As described earlier, the necrotrophic spots and particularly chlorosis spread rapidly, particularly in the fully expanded leaves. At 4 DPI, extensive chlorosis was observed in the *nrpd2a* mutants compared with WT plants (Figure 6A). The extent of leaf maceration in the *nrpd2a* mutants was more severe than that of WT but was significant less than that of the *med18* mutants (Figure 6A). The *nrpd2a* mutant plants also had increased *BcActA* gene transcript levels relative to WT plants, although the increase was lower than in the *med18* mutant plants (Figure 6B). Thus, both symptom development and growth of the fungus confirm that the mutants for both *MED18* and *NRPD2a* genes are more susceptible to *Botrytis*.

NRPD2a is the second largest subunit of both Arabidopsis RNA Pol IV and Pol V. To determine whether the role of NRPD2a in plant disease resistance is linked with its role as a catalytic subunit of the two RNA polymerases, we also tested the phenotypes of the two independent mutants for NRPD1a, the largest subunit of RNA Pol IV. As shown in Figure 6A, at 4 DPI, both mutants for *NRPD1a* developed extensive chlorosis similar to that of the *nrpd2a* mutants, which was substantially more severe than that in WT plants. Consistent with the increased symptom development, the two *nrpd1a* mutants also supported increased fungal growth when compared with WT plants (Figure 6B). Therefore, the mutants for NRPD1a and NRPD2a shared very similar phenotypes of increased susceptibility to the necrotrophic fungal pathogen.

We also analyzed the *med18*, *nrpd2a* mutants and *NRPD2a* overexpression plants for response to a virulent strain of the bacterial pathogen *P. syringae*. As shown in Supplementary Figure 3, when compared to those of WT, the growth of the bacterial pathogen in the *med18* and *nrpd2a* mutants were only slightly increased but the increases were not statistically significant. Overexpression of *NRPD2a* also had little effect on the growth of the bacterial pathogen (Supplementary Figure 3).

### Both MED18 and NRPD2a Affected *Botrytis*-Induced Cell Death and Reactive Oxygen Species Accumulation

**FIGURE 7:**
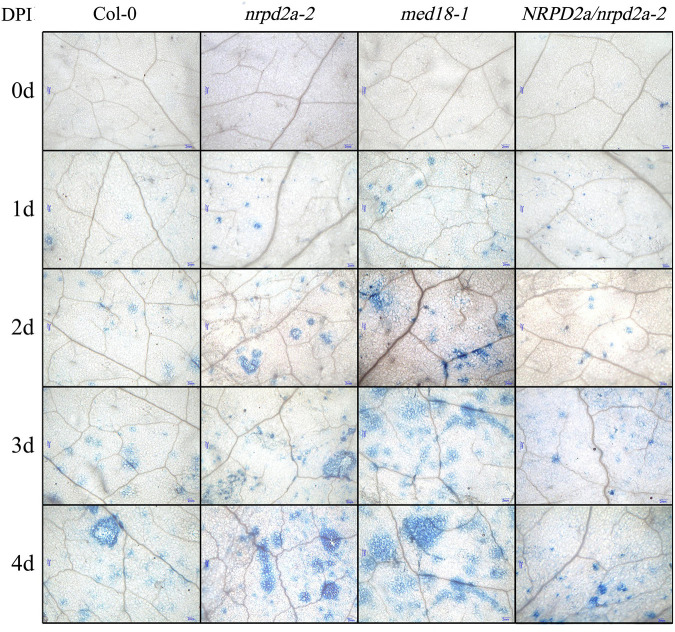
Cell death in *Botrytis*-infected Arabidopsis leaves. Trypan blue staining of leaves of Col-0, *med18* and *nrpd2a* mutants at the indicated days post inoculation (DPI) with *Botrytis*. The experiments were repeated once with similar results.

**FIGURE 8:**
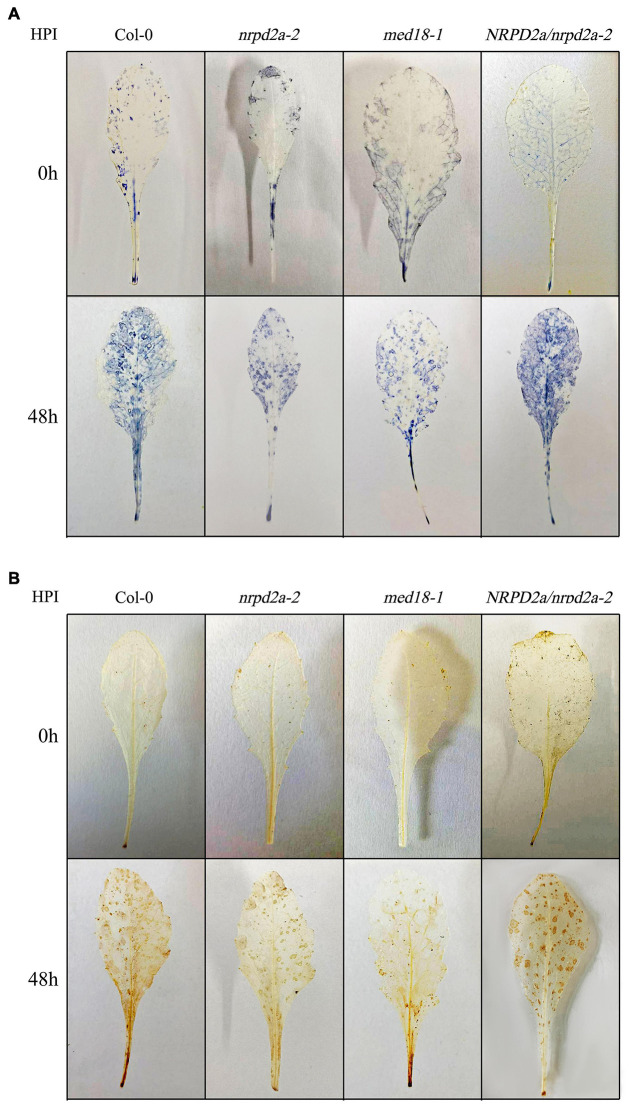
*Botrytis*-induced ROS accumulation. **(A)** Detection of leaf superoxide (O_2_.^–^) by NBT staining. **(B)** Detection of hydrogen peroxide (H_2_O_2_) by DAB staining. Leaves of Col-0, *med18* and *nrpd2a* mutants were collected at the indicated hours post inoculation (HPI) with Botrytis and used for ROS detection using histochemical staining. The experiments were repeated once with similar results.

To further characterize the effect of mutation and overexpression of NRPD2a on plant defense against *Botrytis*, we compared Col-0, *med18* and *nrpd2a* mutants for *Botrytis*-induced cell death using trphan blue staining. As shown in Figure 7, *Botrytis* infection increased cell death in all these plants. However, even at 1 DPI, both the *med18* and *nrpd2a* mutants had substantially more cell death than Col-0 (Figure 7). This difference in the extent of cell death was also observed during the remaining 3 days (Figure 7). Complementation of the *nrpd2a* mutant with the *NRPD2a* gene restored the mutant to the WT level of cell death (Figure 7). We also compared the Col-0, *med18* and *nrpd2a* mutants for *Botrytis*-induced accumulation of superoxide and hydrogen peroxide. As shown in Figure 8, both Reactive Oxygen Species (ROS) levels were elevated at 48 HPI in Col-0 and the mutants when compared to those at 0 HPI. However, the levels of both ROS were substantially lower at 48 HPI in both the *med18* and *nrpd2a* mutants than in Col-0 (Figure 8). Again, expression of *NRPD2a* in the *nrpd2a* mutant restored both ROS levels in the mutant to those in Col-0 (Figure 8). Thus, mutations of *MED18* or *NRPD2a* led to increased susceptibility to *Botrytis*, which was associated with increased cell death but reduced ROS accumulation.

### Both MED18 and NRPD2a Affected *Botrytis*-Induced Jasmonate and Salicylic Acid Accumulation

**FIGURE 9:**
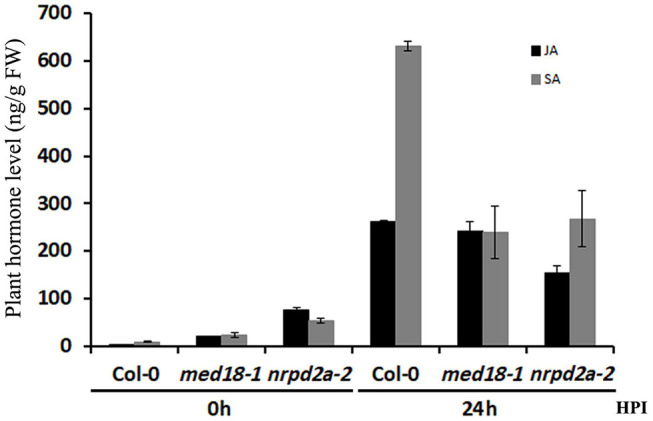
Levels of JA and SA in response to *Botrytis* infection. Leaves of Col-0, *med18* and *nrpd2a* mutants were collected at the indicated hours post inoculation (HPI) with Botrytis and used for JA and SA quantification. The data represent mean ± SD from three biological replicates. The experiments were repeated once with similar results.

We also compared the *med18* and *nrpd2a* mutants with WT for JA and SA levels before and after *Botrytis* infection. As shown in Figure 9, the basal levels of both stress hormones were substantially higher in both the *med18* and *nrpd2a* mutants. At 24 h post *Botrytis* inoculation, the levels of both JA and SA were elevated by about 100- and 70-fold, respectively, in Col-0 WT plants (Figure 9). The levels of both JA and SA were also elevated in both the *med18* and *nrpd2a* mutants after *Botrytis* inoculation. However, although the JA levels in the *med18* mutant were similar to those in Col-0, they were reduced by about 35% in the *nrpd2a* mutant relative to those in Col-0. On the other hand, the levels of SA were reduced by about 50–60% in the *med18* and *nrpd2a* mutants when compared to those in Col-0 at 24 h post *Botrytis* inoculation (Figure 9).

### Transcriptome Profiling of Wild-Type and Mutant Lines in Response to *Botrytis* Infection

**FIGURE 10:**
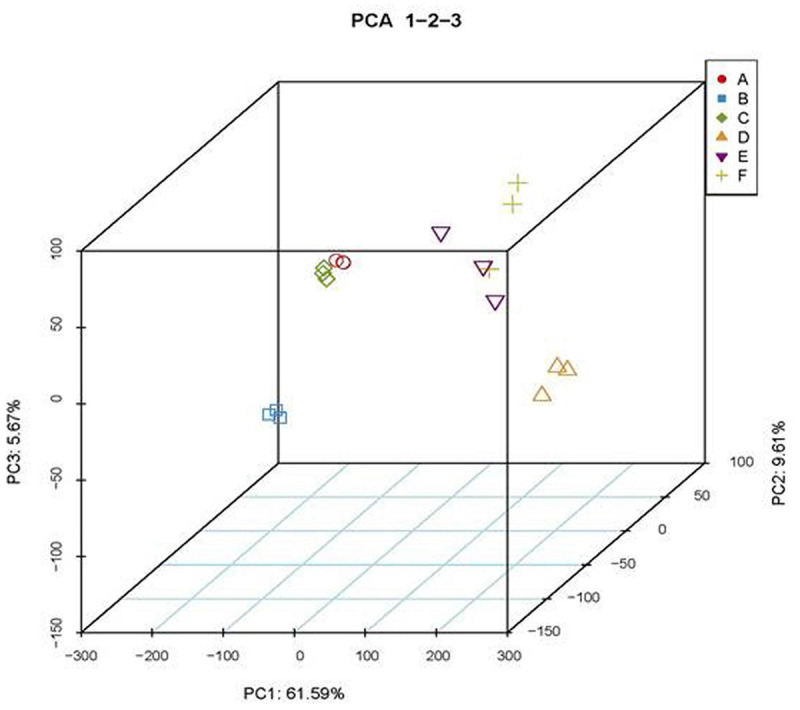
Principal component analysis (PCA) of transcriptome data. A- nrpd2a, 0 HPI; B- med18, 0 HPI; C- Col-0, 0 HPI; D-nrpd2a, 36 HPI; E-med18, 36 HPI; F- Col-0, 36 HPI.

To analyze potential coordinated roles of MED18 and NRPD2a in transcriptional regulation of genes involved in plant immune responses, we compared the transcriptomes of the mutants for the two transcriptional regulators with those of WT prior to and after 36 HPI of *Botrytis*. From 18 samples (three genotypes, two time points, and three biological replicates), we obtained 1017.8 million total filtered reads, of which 931.7 million could be mapped to the Arabidopsis genome assembly, with 40.9–60 million uniquely mapped reads per sample. To obtain the normalized expression values, we calculated FPKMs for all genes in the samples to exhibit distribution of total genes expression. Pearson’s Correlation Coefficient (*r*^2^) analysis confirmed strong correlation between the biological replicates, indicating the high reproducibility of the transcriptome data (Supplementary Table 2). For further quality assessment and exploratory analysis of the transcriptome data, we performed PCA. As shown in Figure 10, the most variation within the data (PC1) accounted for 61.59% of the variance, which was primarily caused by variance between uninfected and pathogen-infected samples. PC2 and PC3 accounted for additional 9.61 and 5.67% of variance, respectively, primarily between uninfected *med18* mutant and the two other genotypes (WT and *nrpd2a*) (Figure 10). On the other hand, the variance between *nrpd2a* mutant and WT was relatively small. Among infected samples, the variance between the *med18* mutant and the two other genotypes was still significant but was substantially reduced when compared to that of uninfected genotypes (Figure 10). Finally, despite their physical interactions and similar roles in resistance to *Botrytis*, the variance in the transcriptome data between the mutants for MED18 and NRPD2a were actually significantly larger than the variance between the mutants and WT (Figure 10). These results suggest that altered expression of specific sets of genes, not global change in transcriptomes, is responsible for the shared role of the two transcriptional regulators in plant immune responses.

### Differentially Expressed Gene Analysis Between Wild-Type and Mutant Lines

**FIGURE 11:**
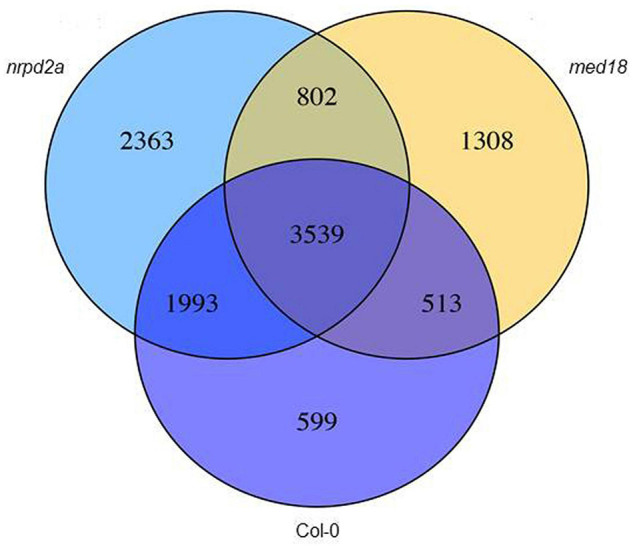
Overlapping in pathogen-regulated DEGs between Col-0 WT and the mutant lines. The numbers of DEGs were obtained from comparison of 0 versus 36 HPI samples from WT, *med18* and *nrpd2a* mutant lines.

In order to gain insights into the roles of MED18 and NRPD2a in pathogen-regulated gene expression, we analyzed DEGs among these three genotypes from the transcriptome profiles. First, we compared pathogen-regulated genes in WT, *med18* and *nrpd2a* mutants by analyzing independently for their respective DEGs through pairwise comparison of 0 and 36 HPI samples. As shown in Figure 11, pathogen infection altered more than 6644, 6162, and 8697 genes in WT, *med18* and *nrpd2a* mutants, respectively. Comparison of the genes that were differentially expressed in each of the three lines showed that there were a large number of shared DEGs (Figure 11), indicating that mutations of *MED18* and *NRPD2a* did not lead to global change in pathogen-regulated gene expression. However, there were substantial numbers of DEGs unique to each line or DEG shared only by the *med18* and *nrpd2a* mutants (Figure 11). These unique DEGs are likely to play an important role in the altered phenotype of the *med18* and *nrpd2a* mutants in resistance to the necrotrophic pathogen.

**FIGURE 12:**
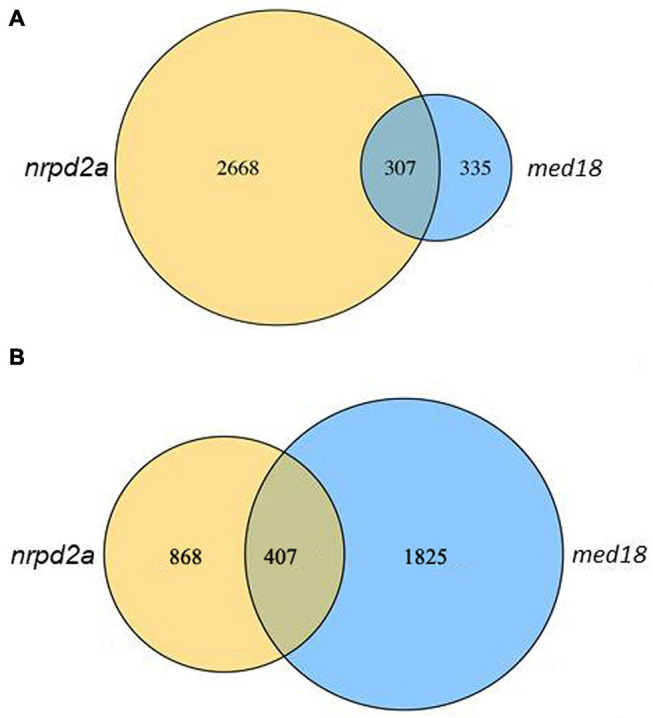
Overlapping in DEGs between WT and the *med18* or *nrpd2a* mutant lines. The numbers of DEGs were obtained from comparison of WT Col-0 vs. the *med18* or *nrpd2a* mutant at 0 **(A)** and 36 **(B)** HPI.

We also analyzed DEGs between WT and the mutant lines at 0 and 36 HPI separately. At 0 HPI, there were 2975 DEGs between WT and the *med18* mutant (Figure 12), consistent with the roles of the mediator subunit in plant growth and development. By contrast, there were only 642 DEGs between WT and the *nrpd2a* mutant at 0 HPI (Figure 12). On the other hand, the number of DEGs between WT and *med18* or *nrpd2a* mutant at 36 HPI was very large (1275 and 2232, respectively) (Figure 12), indicating that both MED18 and NRPD2a transcriptional factors play important roles in pathogen-regulated gene expression in plants. Comparison of the two sets of DEGs between WT and the two mutant lines at 36 HPI revealed that a majority of the DEGs were unique to each mutant line (868 and 1825 for *med18* and *nrpd2a* mutants, respectively) (Figure 12). However, there were also a substantial number of DEGs (407) shared between *med18* and *nrpd2* mutants (Figure 12), which could be co-regulated by MED18 and NRPD2a.

### Gene Ontology and KEGG Analysis of Differentially Expressed Genes Between Wild-Type and Mutant Lines

**FIGURE 13:**
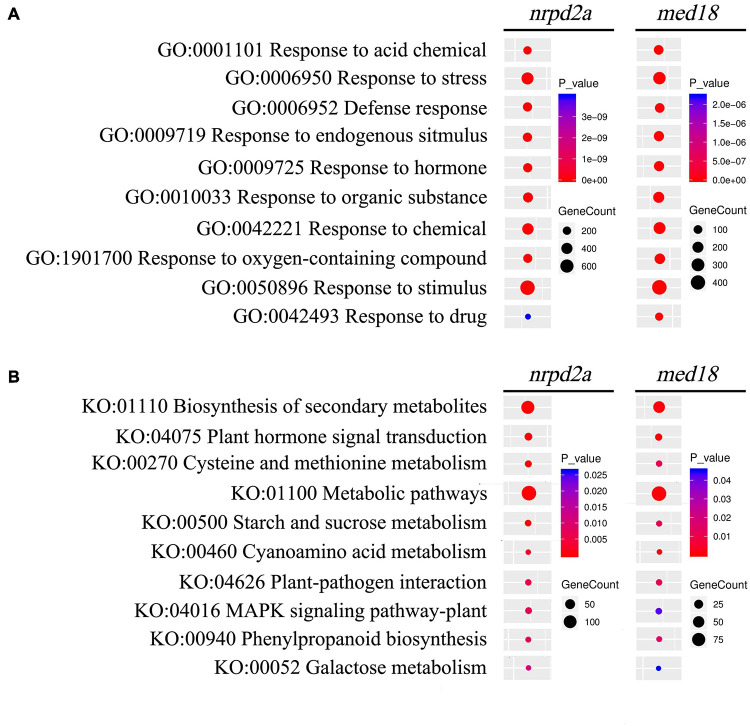
GO and KEGG analysis of genes that are differentially expressed in both the *med18* and *nrpd2a* mutants at 36 HPI. Enrichment was analyzed for “Biological process” GO term **(A)** and KEGG pathways **(B)**. The color of the circles indicates the significance of the term or pathway and their size indicates the number of genes that are associated with that term or pathway.

To understand how MED18 and NRPD2a regulate plant immune responses against *Botrytis*, we performed gene ontology (GO) enrichment analysis to assign biological processes to the identified DEGs between pathogen-infected Col-0 and *med18* or *nrpd2a* mutant line. As shown in Supplementary Figure 4, among the DEGs in the *med18* mutant, those involved in responses to different endogenous and external stimuli including biotic stress, other organisms including bacterium and fungi and hormones were significantly enriched. Genes involved in responses to endogenous and external stimuli were also enriched the DEGs in the *nrpd2a* mutant (Supplementary Figure 5). Interestingly, the DEGs in the *nrpd2a* mutant also included those associated with photosynthesis and responses to a variety of abiotic stresses such as cold, wounding, water, temperature stimuli (Supplementary Figure 5). By comparing the DEGs in the *med18* and *nrpd2a* mutants, we identified common GO terms for biological processes with enriched DEGs from both the *med18* and *nrpd2a* mutants (Figure 13A). These shared GO terms between the DEGs from the two mutants included the biological processes of defense responses and responses to stress, endogenous stimuli, hormone, organic substance and chemical (Figure 13A). Therefore, both MED18 and NRPD2a regulate several biological processes that are closely associated with plant immune responses.

We also performed KEGG pathway enrichment analysis of the DEGs identified at 36 HPI between WT and the *med18* or *nrpd2a* mutant. DEGs with the highest statistical significance in KEGG enrichment in the *med18* mutant have roles in biosynthesis of secondary metabolites, biosynthesis of amino acids, hormone signaling, starch, and sucrose metabolism (Supplementary Figure 6). Other DEGs with significant KEGG enrichment in the *med18* mutant included those involved in plant-pathogen interaction, MAPK signaling. glucosinolate and phenylapropanoid biosynthesis, all of which could potentially play a role in plant defense against necrotrophic pathogens (Supplementary Figure 6 and Figure 13B). Many of these pathways were also enriched in the DEGs identified from the *nrpd2a* mutant, including biosynthesis of secondary metabolism, plant hormone signal and transduction, metabolic pathways, plant-pathogen interaction, MAPK signaling pathway-plant and phenylpropanoid biosynthesis (Figure 13B). However, unlike in the *med18* mutant, DEGs for some of the pathways in biosynthesis of specific amino acids were not enriched but DEGs for biosynthesis were significantly enriched in the *nrpd2a* mutant (Supplementary Figures 6, 7 and Figure 13B). Taken together, the KEGG analysis supports the finding from the GO enrichment analysis that a substantial number of genes involved in defense-related pathways were altered in expression in both the *med18* and *nrpd2a* mutants in response to infection by *Botrytis*.

### Co-regulation of Defense-Related Genes by MED18 and NRPD2a

**FIGURE 14:**
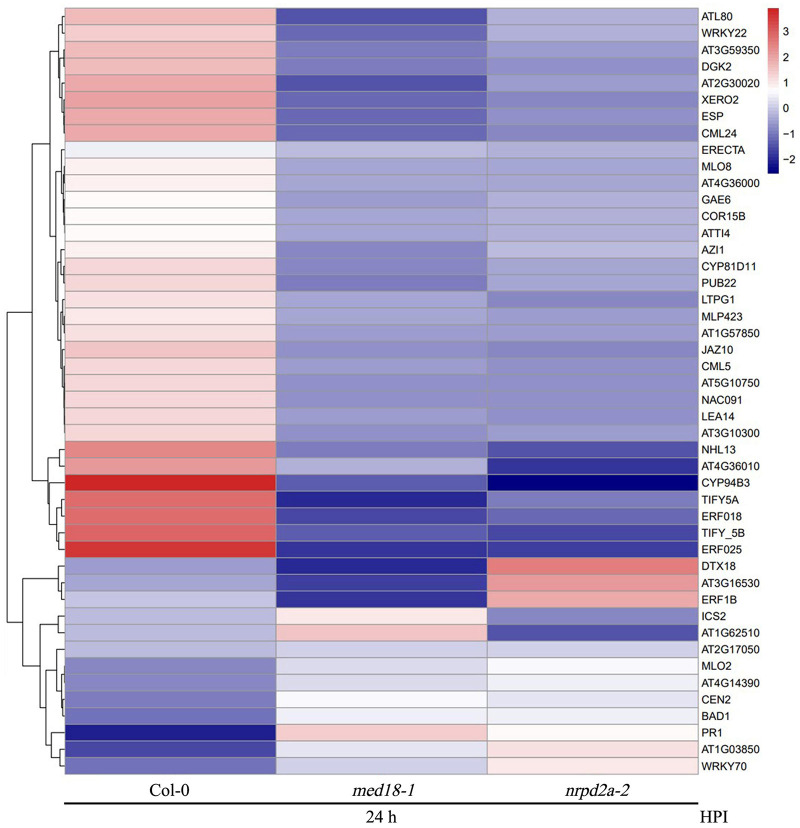
Heatmap of defense-related DEGs in the *med18* and *nrpd2a* mutants at 36 HPI compared to WT. red: upregulated DEGs; blue: downregulated DEGs. The color bar indicates the values of fold change.

To further understand the role of MED18 and NPRD2a in defense against *Botrytis*, we analyzed 46 DEGs that were enriched in the GO term of defense responses and KEGG pathway of plant-pathogen interactions and were altered in expression in both the *med18* and *nrpd2a* mutants (Figure 14). Among these DEGs are three genes encoding disease resistance proteins (AT1G57850, AT2G17050 and AT5G10750). In addition, two genes encoding proteins similar to the MLO resistance protein (AT1G11310 and AT2G17480) were among the 46 DEG (Figure 14). Altered expression of these disease resistance genes could lead to defense responses that impact resistance to invading pathogens. The second group of genes included those involved in SA biosynthesis (AT1G18870, ICS2), signaling (WRKY70 and BDA1) and SA response (PR1) (Figure 14). Both WRKY70 and BDA1 are responsive to SA and act as positive regulators of SA signaling (Li et al., 2004; Yang et al., 2012; Li et al., 2017). *PR1* gene is a useful molecular marker for SA-dependent systemic acquired resistance (Durrant and Dong, 2004; Conrath, 2006). Interestingly, *WRKY70*, *BDA1*, and *PR1* gene were all substantially elevated in both the *med18* and *nrpd2a* mutants (Figure 14), indicating enhanced SA signaling in the mutants. The third group of genes encodes proteins involved in JA metabolism and signaling. Among them are AT3G48520, which encodes CYP94B3, an jasmonoyl-isoleucine-12-hydroxylase that catalyzes the formation of 12-OH-JA-Ile from JA-Ile (Koo et al., 2011) and the genes encoding JAZ7, 8 and 10, transcriptional repressors of JA signaling (Cerrudo et al., 2012; Shyu et al., 2012; Yu et al., 2016). These JA metabolic and signaling genes are often up-regulated by wounding and JA (Cerrudo et al., 2012; Shyu et al., 2012; Yu et al., 2016) but were all down-regulated in the *med18* and *nrpd2a* mutants at 36 HPI (Figure 14), suggesting compromised JA signaling in the mutants. Taken together, these results strongly suggest that mutations of *MED18* and *NRPD2a* led to enhanced SA signaling but compromised JA signaling in response to infection by a necrotrophic fungal pathogen.

Both ABA and ethylene (ET) play roles in plant immunity. MED18 also regulates plant abiotic stress responses through interaction with ABA INSENSITIVE 4 (Lai et al., 2014). There was also significant difference in the accumulation of ROS in both the *med18* and *nrpd2a* mutants after *Botrytis* infection (Figure 8). Therefore, we also analyzed the differential expression of ABA-, ET-, and ROS-related genes in the *med18* and *nrpd2a* mutants. First, we surveyed that the 46 co-regulated DEGs associated with defense responses and plant–pathogen interactions and discovered several to be associated with ABA and ET signaling and responses. One of these three genes is AT2G30020, which encoding a PP2C with a role in ABA signaling (Figure 14). The other two genes encoding ERF018 (AT1G74930) and ERF025 (AT5G52020) possibly involved in ET signaling were also downregulated in the *med18* and *nrpd2a* mutants (Figure 14). Secondly, we have also examined a range of genes known to be involved in the production and signaling of ABA, ET, and ROS for their differential expression in the *med18* and *nrpd2a* mutants. As shown in Supplementary Figure 8, expression of some of the ABA-, ET-, and ROS-related genes was substantially altered in *med18*, *nrpd2a* or both mutants, particularly after *Botrytis* infection. Specifically, after *Botrytis* infection, three of the four ABA-related genes were modestly elevated in the *med18* mutant but one of them elevated in the *nrpd2a* mutant when compared to Col-0 (Supplementary Figure 8A). On the other hand, expression of three of the four ET-related genes (*ERF1*, *ORA59*, and *ERF5*) were substantially elevated in infected *nrpd2a* mutant but slightly reduced in the *med18* mutant (Supplementary Figure 8B). For ROS-related genes, expression of two genes encoding ROS-generating NADPH oxidases (RbohD and F) were substantially reduced in infected *med18* mutant but was either not altered (RbohD) or even slightly increased (RbohF) in the *nrpd2a* mutant) (Supplementary Figure 8C). Expression of *BIK1*, which has an important role in defense signaling leading to ROS generation, was elevated in the *nrpd2a* mutant but unaltered in the *med18* mutant (Supplementary Figure 8C). On the other hand, expression of *TRX-h5*, which encodes a cytosolic thioredoxin involved in oxidative stress responses, was modestly elevated in both the *med18* and *nrpd2a* mutants following *Botrytis* infection relative to that in Col-0 (Supplementary Figure 8C). Taken together, some ABA-, ET, and ROS-related genes were altered in expression in the *med18* and *nrpd2a* mutants but not necessarily in a coordinated manner.

Plant-specific Pol IV and V transcribe non-coding sequences and, therefore, their roles in the regulation of Pol II-dependent transcription of protein-coding genes are indirect, through siRNAs from Pol IV- and Pol V-transcribed RNA precursors from repetitive DNA sequences and transposable elements. To determine whether transposable elements play a role in altered expression of defense-related genes in the *nrpd2a* mutant, we examined whether the loci of these genes contain transposable elements in close proximity. Interestingly, among the 46 defense-related DEGs, 22 contain at least two annotated transposable elements within or immediately adjacent to the loci (Figure 14). Among these 22 genes, however, a majority of them (17) were down-regulated in pathogen-infected *nrpd2a* mutant. The five up-regulated defense-related genes with nearby transposons encode the GRXS13 (a glutaredoxin), PR1, a disease resistance protein, a lectin-like protein and an ankyrin repeat protein, which is highly similar to ACD6 (ACCELERATED CELL DEATH 6) involved in plant defense (Lu et al., 2005; Figure 14). It would be of interest to analyze with the transposable elements within or immediately adjacent to the genes play any role in their expression.

## Discussion

Mediator is a conserved transcriptional regulatory complex that regulates transcription by mediating interactions between transcriptional activator proteins and RNA Pol II. Arabidopsis MED18 is a Mediator subunit with important roles in a broad spectrum of biological processes including flowering, hormone signaling and plant immunity (Lai et al., 2014; Liao et al., 2016). The multifunctional nature of the Mediator subunit is mediated through interactions with multiple transcription factors (Lai et al., 2014; Liao et al., 2016). Using yeast two-hybrid screens, we discovered that Arabidopsis MED18 also interacted with NRPD2a, the second largest subunit of the nuclear Pol IV and V (Figure 1). MED18-NRPD2a interaction was also confirmed in plant cells using both BiFC (Figure 2) and Co-IP (Figure 3). Plant-specific Pol IV and V each contain 12 subunits that are either identical or paralogous to the 12 subunits of Pol II. As a result, plant Pol II, IV and V could potentially interact with same proteins through their common subunits. However, NRPD2a is identified only in Pol IV and V and, therefore, its interaction with Med18 subunit raises a strongly possibility that the Mediator complex may regulate transcription not only by Pol II, but also by Pol IV and V in plants. This possibility is consistent with the previous finding that Mediator is involved in transcriptional silencing of repeats and transposons, which requires plant-specific Pol IV and V (Kim et al., 2011). The same study also revealed that Pol V occupancy at specific silenced loci of repeats and transposons was reduced in the Arabidopsis mutant for the Mediator subunit MED20a (Kim et al., 2011). Furthermore, a previously reported proteomic analysis found that the plant-specific Mediator subunit MED36 co-purified with the largest subunit of Pol V (Huang et al., 2009). Taken together, these discoveries challenge the paradigm that the Mediator complex is involved only in regulation of transcription by Pol II.

The Mediator subunit MED18 and the Pol IV and V subunit NRPD2a interact not only physically but also functionally. First, both MED18 and NRPD2a are induced by Botrytis infection (Figure 4) and are positive regulators of plant resistance to the necrotrophic fungal pathogen *Botrytis* based on the phenotypes of their mutants and overexpression lines (Figures 5, 6; Lopez et al., 2011; Lai et al., 2014). The important role of NRPD2a in plant immunity against the necrotrophic pathogen is not a unique function of this specific subunit of Pol IV and V because mutants for NRPD1a were also compromised in resistance against *Botrytis* (Figure 6). Secondly, in addition to enhanced disease symptoms and Botrytis growth (Figures 5, 6), both *med18* and *nrpd2a* mutants displayed increased cell death (Figure 7), but intriguingly, reduced ROS accumulation after Botrytis infection (Figure 8). Increased cell death in the mutants was likely associated with their increased susceptibility to the necrotrophic pathogen, which causes cell death at very early stages of infection to extract nutrients from dead or dying host cells. Despite increased cell death, both the med18 and nrdpr2a mutants had reduced ROS levels, suggesting that both MED18 and NRPD2a may both positive regulate ROS accumulation after *Botrytis* infection. Previously, increased ROS production has been linked with increased plant resistance to *Botrytis* (Asselbergh et al., 2007; Sivakumaran et al., 2016). Thirdly, we performed transcriptome profiling of Arabidopsis WT, *med18* and *nrpd2a* mutants and found that at both 0 and 36 HPI, there was substantial overlapping between the genes whose expression was affected in the *med18* versus in *nrpd2a* mutants (Figure 12). Thus, MED18 and NRPD2a may coordinate through physical interaction to regulate transcription of genes important for fitness and defense responses.

MED18- and NRPD2a-coregulated defense-related genes are involved in a wide spectrum of biological processes and pathway including stress responses, signaling, primary and secondary metabolism (Figure 13). They also include genes directly involved in plant–pathogen interactions such as those encoding PR proteins, including PR1, whose gene expression was drastically elevated in both the *med18* and *nrpd2a* mutants when compared to that in WT at 36 HPI (Figure 14). Induced *PR1* expression is considered to be one of the most reliable molecular markers for SA-dependent systemic acquired resistance (SAR) (Durrant and Dong, 2004). The SA levels were elevated substantially in uninfected *med18* and *nrpd2a* mutants but were actually lower in the mutants after *Botrytis* infection (Figure 9). Therefore, elevated expression of SA-regulated *PR* genes in the *med18* and *nrpd2a* mutants could be mediated by elevated basal SA levels and increased SA signaling upon *Botrytis* infection, which is known to have a negative feedback effect on SA production in the mutants (Durrant and Dong, 2004). SAR is often induced in resistant plants upon recognition of avirulent effector proteins by the disease resistance (R) proteins and is associated with increased SA biosynthesis and signaling (Durrant and Dong, 2004). Interestingly, MED18- and NRPD2a-coregulated defense-related genes also include several SA-responsive genes including WRKY70 and BDA1, which play a positive role in SA signaling (Figures 9, 14; Li et al., 2004, 2017; Yang et al., 2012). R protein-mediated immunity including hypersensitive cell death and SA signaling play an important positive role in defense against biotrophic pathogens, which rely on long-term feeding relationship with the living plant host cells. Necrotrophic pathogens such as *Botrytis* kill host cells before colonizing them (Mengiste, 2012). Although effective against biotrophic pathogens, the hypersensitive response is actually promoted by necrotrophic pathogens and facilitates their infection. In Arabidopsis, resistance to necrotrophic pathogens depends on JA and ET signaling and synthesis of the phytoalexin camalexin. SA can often antagonize JA signaling pathway to promote susceptibility to necrotrophic pathogens (Zheng et al., 2006). A number of JA- and ET-signaling genes were down-regulated in both the *med18* and *nrpd2a* mutants at 36 HPI of *Botrytis* (Figure 14). Therefore, both MED18 and NRPD2a may promote plant resistance to necrotrophic pathogens by acting as negative regulators of SA signaling but positive regulators of JA and ET signaling during response to the fungal infection.

The plant-specific multisubunit Pol IV and V play important roles in RNA-directed chromatin modification including DNA methylation that silences transposable elements as a defense mechanism for maintaining genome stability in both plants and mammals (Ream et al., 2014). A number of studies have shown that transposable elements and other non-coding sequences can affect gene expression associated with plant immunity (Alonso et al., 2019). Plant R genes often form gene clusters in the genome that contain repetitive sequences and transposons (Meyers et al., 2003). Transposons inserted in the promoter regions often regulate neighboring genes by changing their epigenetic states. Studies on cytosine DNA methylation in rice and Arabidopsis indicates that when transposons are within or in proximity to stress-inducible genes, they play a critical role in responsiveness to environmental stress cues (Secco et al., 2015). Interestingly, a number of R genes and other defense-related genes such as ICS2 coregulated by MED18 and NRPD2a contain transposable elements in their promoter regions (Figure 14) and, therefore, their altered expression in *Botrytis* infected *med18* and *nrpd2a* mutants could be mediated through altered DNA methylation and other chromatin state in their promoters as affected by neighboring transposons or other repetitive sequences. Further research will be necessary to confirm the epigenetic nature of the functional interaction between MED18 and NRPD2a in their regulation of plant immunity and expression of defense-related genes.

It is known that Mediator plays an important role in non-coding RNA production. Previously, it has been shown that Mediator is required for microRNA (miRNA) biogenesis by recruiting Pol II to promoters of miRNA genes (Kim et al., 2011). In Mediator mutants, several well-characterized heterochromatic loci are de-repressed and that Mediator promotes Pol II-mediated production of long non-coding scaffold RNAs, which serve to recruit Pol V to these loci (Kim et al., 2011). In addition, Arabidopsis Mediator subunit MED19a directly interacts with a long-non-coding RNA, designated ELF18-INDUCED LONG-NONCODING RNA1 (ELENA1) (Seo et al., 2017). This interaction affects enrichment of MED19a on the PR1 promoter (Seo et al., 2017). These studies indicate that Mediator has a broader role not only in Pol II-mediated production but also in the action of regulatory non-coding RNAs. The demonstrated physical and functional interaction of MED18-NRPD2a interaction further expands the roles of Mediator in the epigenetic regulation of gene expression and promotion of genome stability.

In summary, we have demonstrated that Arabidopsis Mediator subunit MED18 interacts with NRPD2a, the second largest subunit of Pol IV and V (Figures 1–3). We have provided further evidence for potential functional interaction between MED18 and NRPD2a through analysis of their role in resistance to the necrotrophic fungal pathogen *Botrytis* and in pathogen-regulated gene expression. MED18 and NRPD2a regulate expression of genes involved in a broad spectrum of biological processes and pathways. MED18- and NPRD2a-coregulated genes also include those encoding R proteins, SA biosynthetic ICS2 enzyme and PR proteins, whose enhanced expression in the *med18* and *nrpd2a* mutants may contribute to the altered phenotype of the mutants in resistance to the necrotrophic fungal pathogen *Botrytis*. The discovery of MED18-NRPD2a interaction also raises the possibility that some of the broad roles of Mediator are mediated directly by plant-specific Pol IV and V.

## Data Availability Statement

The datasets presented in this study can be found in online repositories. The names of the repository/repositories and accession number(s) can be found below: www.ncbi.nlm.nih.gov/, PRJNA717217.

## Author Contributions

YZ and GX: conceptualization and funding acquisition. YZ and WX: methodology. YZ and CS: performance of a majority of research work. WF, XG, and ZQ: contribution to some research work. YZ: data analysis and writing. All authors read and agreed to the published version of the manuscript.

## Conflict of Interest

The authors declare that the research was conducted in the absence of any commercial or financial relationships that could be construed as a potential conflict of interest.

## Publisher’s Note

All claims expressed in this article are solely those of the authors and do not necessarily represent those of their affiliated organizations, or those of the publisher, the editors and the reviewers. Any product that may be evaluated in this article, or claim that may be made by its manufacturer, is not guaranteed or endorsed by the publisher.

## Footnotes

1
http://bioinformatics.sdstate.edu/go/

2
https://bioinfogp.cnb.csic.es/tools/venny/index.html
