# Supplementary material for: Arabidopsis MED18 Interaction With RNA Pol IV and V Subunit NRPD2a in Transcriptional Regulation of Plant Immune Responses
Source: Front Plant Sci. 2021 Oct 6;12:692036. doi: 10.3389/fpls.2021.692036 (PMC8527527; doi:10.3389/fpls.2021.692036)
Supplement: Supplementary file 1 [file Data_Sheet_1.docx]

Supplementary Material

# Supplementary Figures and Tables

## Supplementary Figures


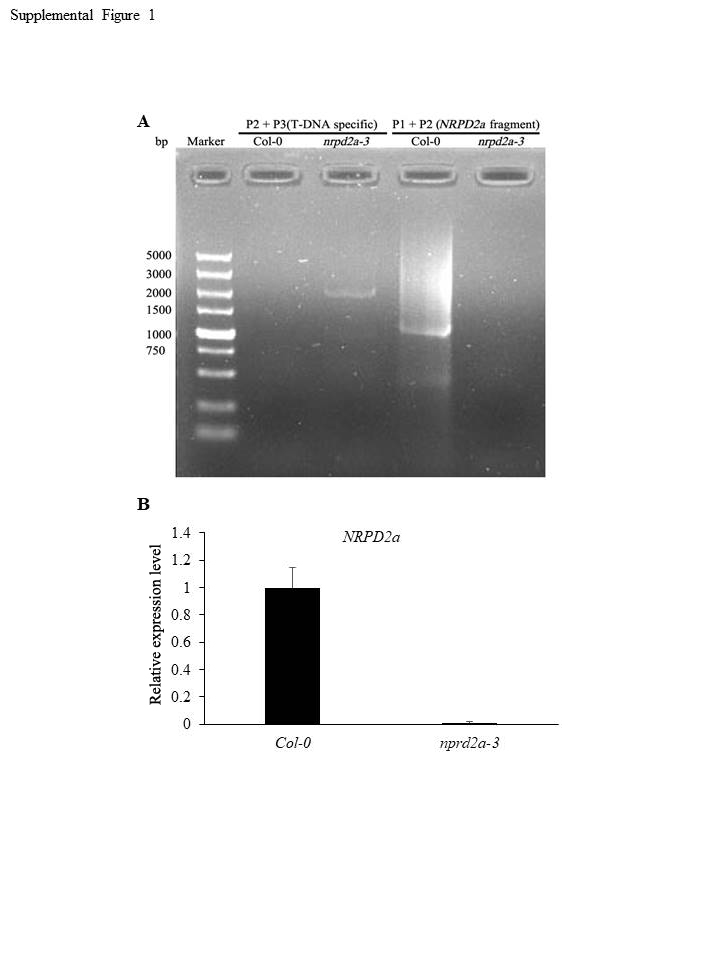


**Supplementary Figure 1.** Identification and expression analysis of the *nrpd2a-3* mutant. (A) PCR identification of homozygous *nrpd2a-3* T-DNA insertion mutant. PCR reaction using primers flanking the T-DNA insertion site (P1 + P2) amplified a fragment of ~1,000bp from Col-0 but not from the *nrpd2a-3* mutant. PCR reaction using a primer in the *NRPD2a* gene adjacent to the T-DNA insertion site in combination with primer for the left border sequence of inserted T-DNA amplified a fragment of ~2,000 bp from the nrpd2a-3 mutant but not from Col-0. (B) Relative expression levels of *NRPD2a* in Col-0 and *nrpd2a-3* determined by RT-qPCR using gene-specific primers. The data represent mean and SD calculated from three independent biological replicates.


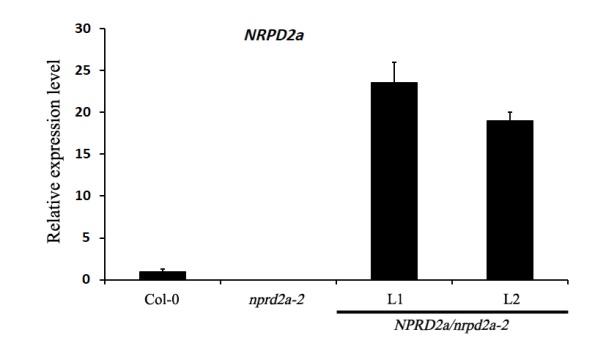


**Supplementary Figure 2.** Expression of *NRPD2a* of the *NRPD2a*-overexpression lines. Relative expression levels of *NRPD2a* in Col-0 and two transgenic lines of overexpression plants (L1 and L2) were determined by RT-qPCR using gene-specific primers. The data represent mean and SD calculated from three independent biological replicates.


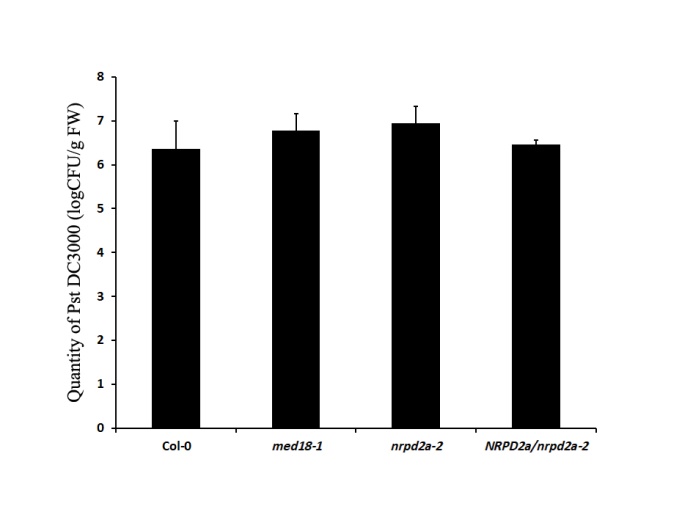


**Supplementary Figure 3.** Growth of bacterial pathogen *P. syringae*. Col-0, mutants and overexpression plants were infiltrated with a suspension of *P. syringar* pv *tomato* DC3000 (OD600 = 0.0002 in 10 mM MgCl2). Leave samples were taken at 3 DPI to determine the bacterial growth, means of colony-forming units (cfu) and SD were calculated from at least ten leaves for each genotype.


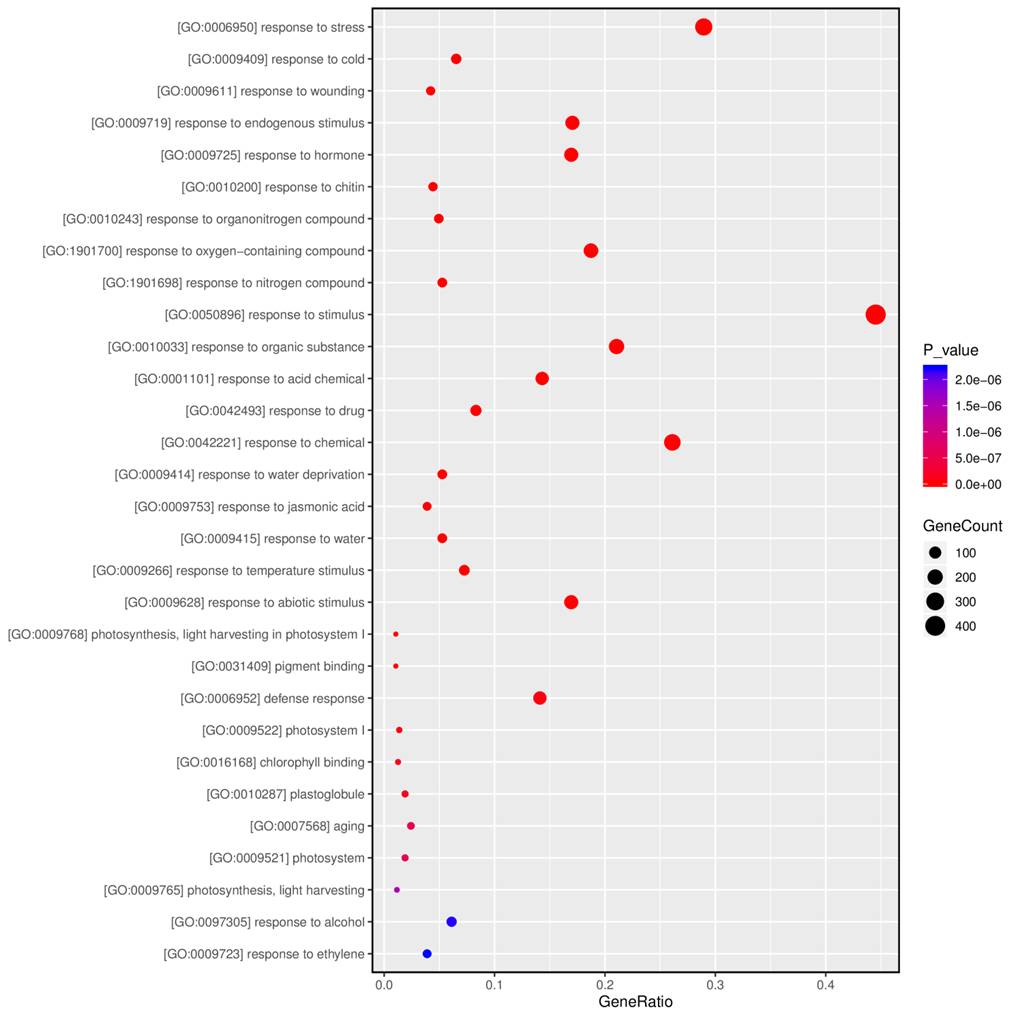


**Supplementary Figure 4.** GO enrichment among genes that are differentially expressed in the *med18* mutant compared to Col-0 at 36 HPI. Enrichment was analyzed for “Biological process” GO term. The color of the circles indicates the significance of the term and their size indicates the number of genes that are associated with that term.


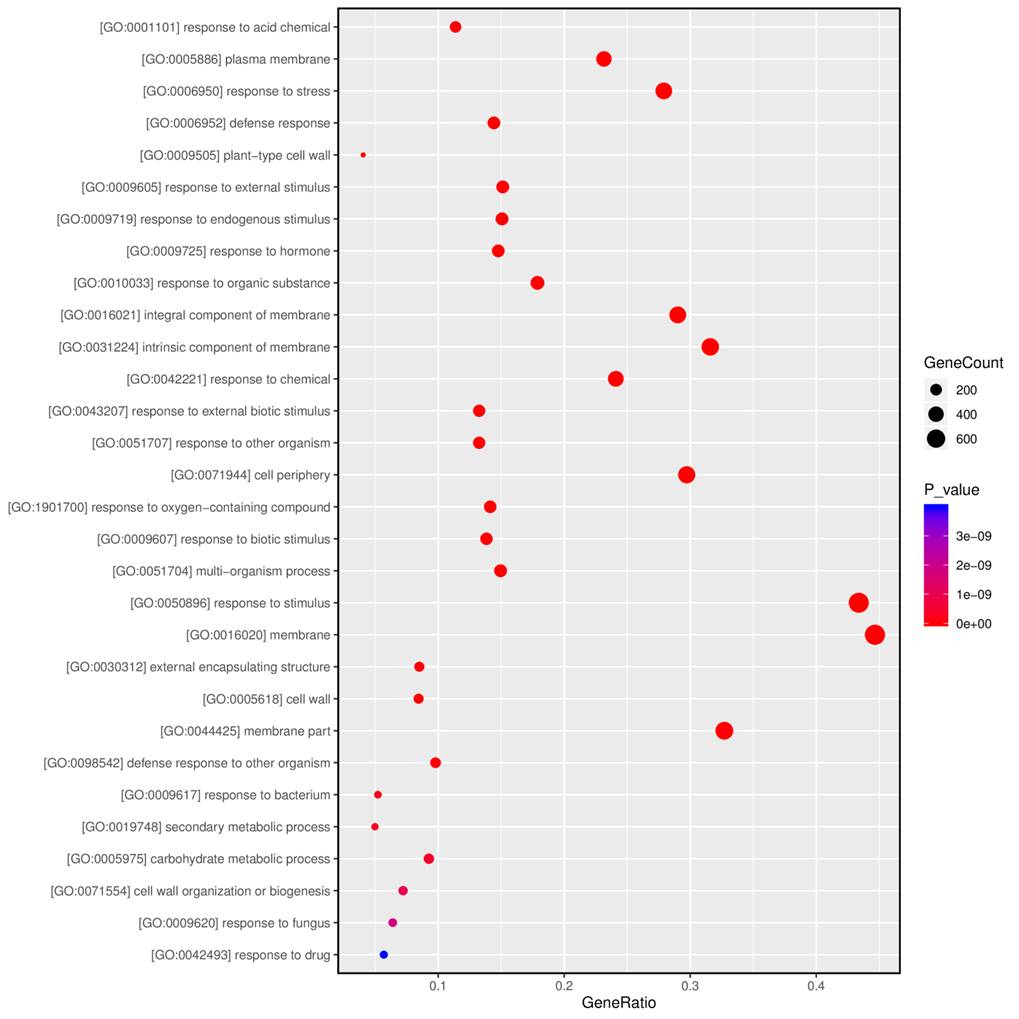


**Supplementary Figure 5.** GO enrichment among genes that are differentially expressed in the *nrpd2a* mutant compared to Col-0 at 36 HPI. Enrichment was analyzed for “Biological process” GO term. The color of the circles indicates the significance of the term and their size indicates the number of genes that are associated with that term.


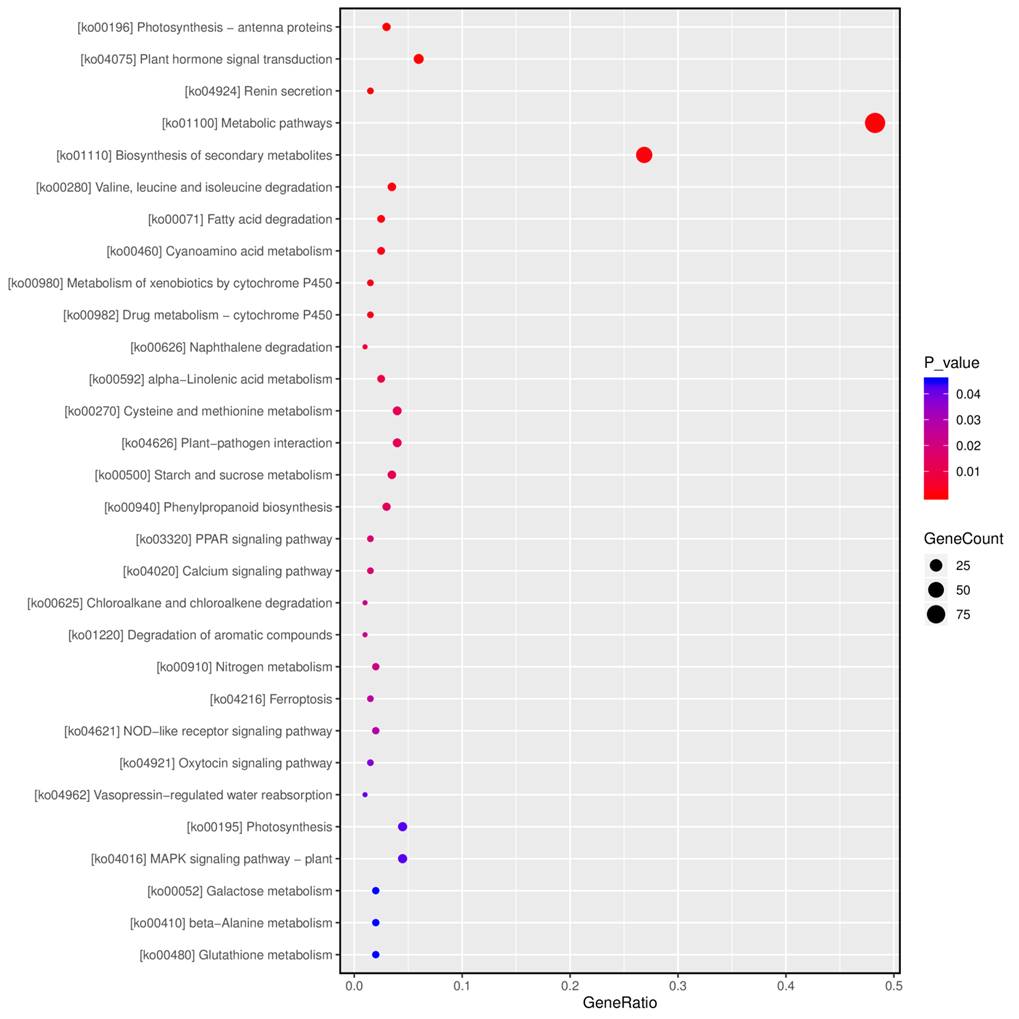


**Supplementary Figure 6.** KEGG pathway enrichment among genes that are differentially expressed in the *med18* mutant compared to Col-0 at 36 HPI. The color of the circles indicates the significance of the pathway and their size indicates the number of genes that are associated with that pathway.


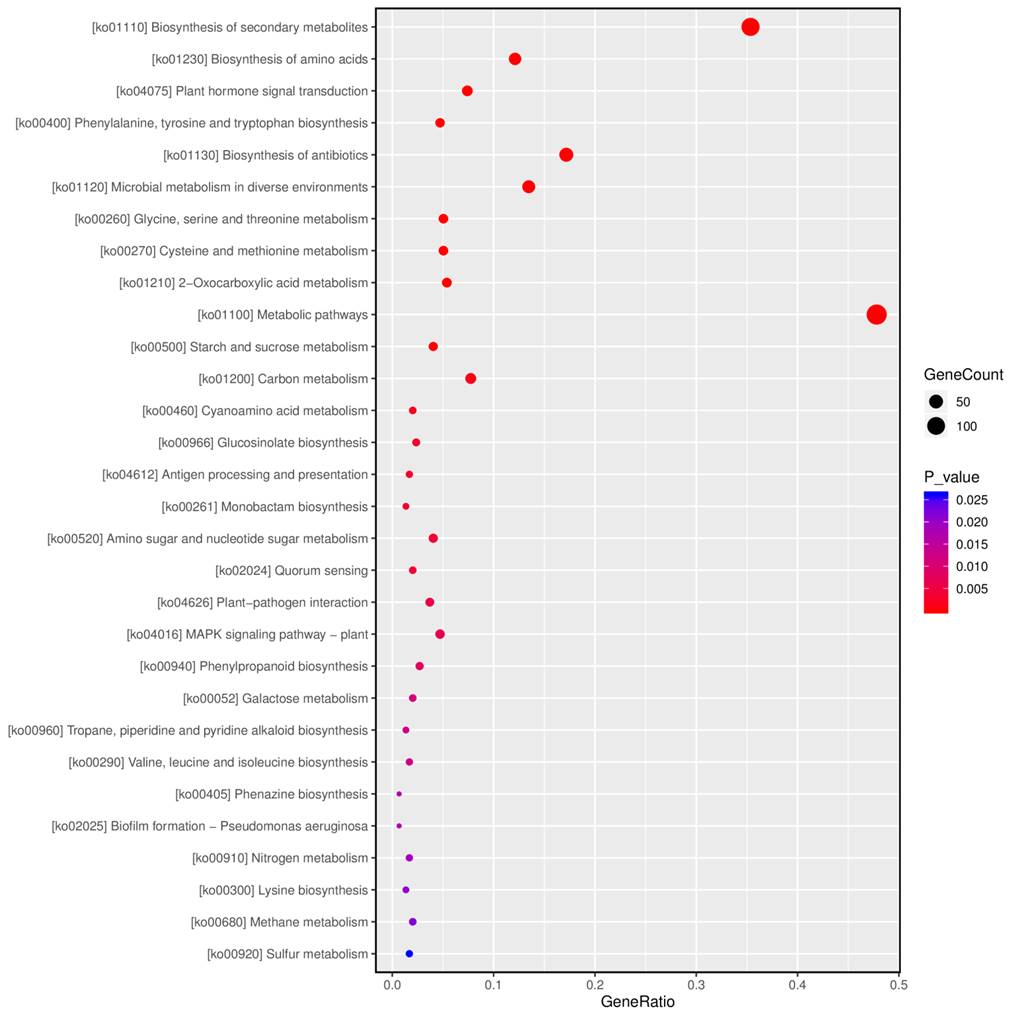


**Supplementary Figure 7.** KEGG pathway enrichment among genes that are differentially expressed in the *nrpd2a* mutant compared to Col-0 at 36 HPI. The color of the circles indicates the significance of the pathway and their size indicates the number of genes that are associated with that pathway.


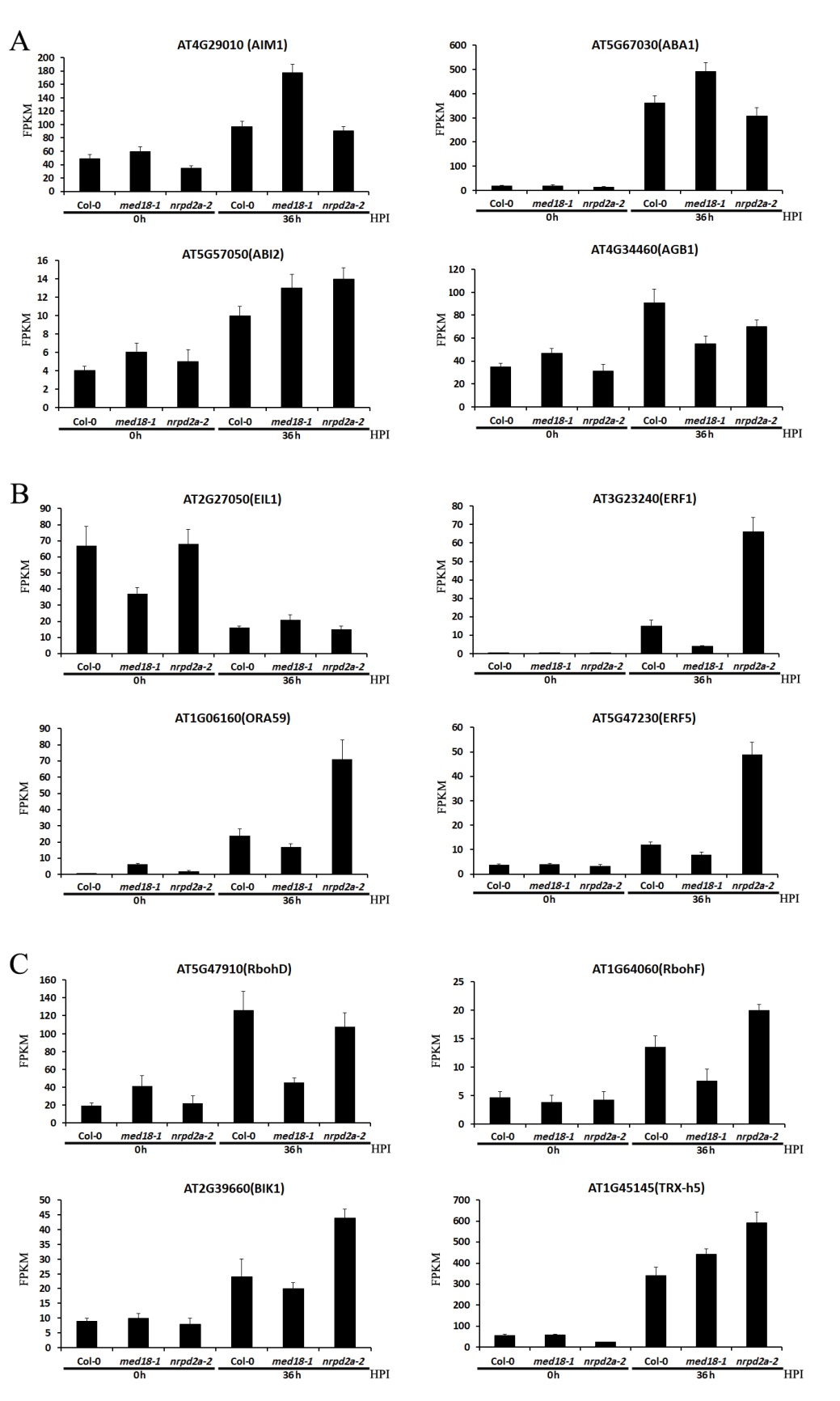


**Supplementary Figure 8.** Transcript levels of ABA- (A), ET- (B) and ROS-related (C) genes in Col-0, *med18-1*, *nrpd2a-2* and *NRPD2a*-overexpression lines from the transcriptome profiling. The data represent means of FPKMs and SD calculated from three independent biological replicates.

**1.2 Supplementary Tables**

Supplementary Table 1. Primers used in this study

| **Primer** | **Sequence (5’ to 3’)** | **Note** |
| --- | --- | --- |
| PCR | | |
| *nrpd2a-3* (WiscDsLoxHs048_04C) homozygous lines identification | | |
| P1 | TCTTAGGCTCTGGGACAGATG |  |
| P2 | GACTGCAGAAACAGTGATCGG |  |
| P3 | AACGTCCGCAATGTGTTATTAAGTTGTC | WiscDsLox T-DNA specific primer |
| *MED18* fused in pBD-GAL4 plasmid for yeast-two hybridization | | |
| P4 | ATGAATCCATGTCAAATGGAGTGTGTGGT |  |
| P5 | ATGTCGACTTACAATGTAGTACCTCCTC |  |
| *NRPD2a*, *NRPD2aCTD* and *NTD* fused in pFGC-N-YFP for BiFC | | |
| P5 | TCTCTCTCGAGCTTTCGCGAGCTCATGCCAGATATGGACATTG | For *NRPD2a* and *NRPD2a NTD* |
| P6 | CATGGTGGCGATGGATCTTCTAGAGAACAGCACTTCTTTTTTAAG | For *NRPD2a NTD* |
| P7 | TCTCTCTCGAGCTTTCGCGAGCTCATGAATGGCCAGAACGCAATTG | For *NRPD2a CTD* |
| P8 | CATGGTGGCGATGGATCTTCTAGAGCATAGCTTGGTGTCGAAG | For *NRPD2a* and *NRPD2a CTD* |
| *MED18* fused in pFGC-C-YFP for BiFC | | |
| P9 | TCTCTCTCGAGCTTTCGCGAGCTCATGTCAATGGAGTGTGTG |  |
| P10 | GGTACCGGATCCCTCGAGTCTAGACAATGTAGTACCTCCTCC |  |
| *NRPD2a* fused in pBA-Myc for Co-IP | | |
| P11 | CGGGGGACTCTAGAGGATCTCGAG ATGCCAGATATGGACATTG |  |
| P12 | TTTGAATCGATACCGTCGAGACGTCTGTCAGCATAGCTTGGTGTCG |  |
| *NRPD2a* CDS for over-expression | | |
| P13 | AGGACTTGAATTCGGTACCCCGGATGCCAGATATGGACATTG |  |
| P14 | CGCGTCCTAGGCTACGTAGGATCCATCAGCATAGCTTGGTGTC |  |
| qPCR | | |
| *MED18* | | |
| P15 | ACAGCATGTTGAAGCTCTTG |  |
| P16 | TTCTGATGACACAACTCCAAG |  |
| *NRPD2a* | | |
| P17 | TATCCGAACCGAAGAAAT |  |
| P18 | AGAAACGACTCCGAGCAA |  |
| Arabidopsis *Actin2* | | |
| P19 | GCTGTTGACTACGAGCAGGA |  |
| P20 | ACAAACGAGGGCTGGAACAA |  |
| Botrytis *Actin* | | |
| P21 | TGGTCGTGATTTGACTGATT |  |
| P22 | GACTGGCGGTTTGGATTTCT |  |

Supplementary Table 2. Pearson’s Correlation Coefficient (r2) of biological replicates*

| **Sample1** | **Sample2** | **r^2^** | **Sample1** | **Sample1** | **r^2^** | **Sample1** | **Sample1** | **r^2^** |
| --- | --- | --- | --- | --- | --- | --- | --- | --- |
| A1 | A2 | 0.96 | B2 | B3 | 0.96 | D1 | D3 | 0.95 |
| A1 | A3 | 0.97 | C1 | C2 | 0.96 | D2 | D3 | 0.94 |
| A2 | A3 | 0.96 | C1 | C3 | 0.95 | E1 | E2 | 0.91 |
| B1 | B2 | 0.96 | C2 | C3 | 0.95 | E1 | E3 | 0.94 |
| B1 | B3 | 0.94 | D1 | D2 | 0.92 | E2 | E3 | 0.95 |
| F1 | F2 | 0.86 | F1 | F3 | 0.93 | F2 | F3 | 0.93 |

*Three biological replicated for the following genotype/treatment: A, *nrpd2a*, 0 HPI; B, *med18*, 0 HPI; C, Col-0, 0 HPI; D, *nrpd2a*, 36 HPI; E, *med18*, 36 HPI; F, Col-0, 36 HPI.
